# Changes in VO_2_ Kinetics After Elevated Baseline Do Not Necessarily Reflect Alterations in Muscle Force Production in Both Sexes

**DOI:** 10.3389/fphys.2019.00471

**Published:** 2019-04-25

**Authors:** Paulo Cesar do Nascimento Salvador, Lisa Schäfer, Bruno Grassi, Luiz Guilherme Antonacci Guglielmo, Benedito Sérgio Denadai

**Affiliations:** ^1^Physical Effort Laboratory, Sports Center, Federal University of Santa Catarina, Florianopolis, Brazil; ^2^Leonardo da Vinci University/Uniasselvi, Indaial, Brazil; ^3^School of Sport and Service Management, University of Brighton, Eastbourne, United Kingdom; ^4^Exercise Physiology Laboratory, Department of Medicine, Università Degli Studi di Udine, Udine, Italy; ^5^Human Performance Laboratory, São Paulo State University, Rio Claro, Brazil

**Keywords:** motor unit recruitment, muscle fatigue, O_2_ delivery, oxidative phosphorylation, elevated baseline, VO_2_ kinetics

## Abstract

A link between muscle fatigue, decreased efficiency and the slow component of oxygen uptake (VO_2_sc) has been suggested. However, a cause-effect relationship remains to be elucidated. Although alterations in VO_2_ kinetics after elevated baseline work rate have previously been reported, to date no study has observed the effect on muscle force production (MFP) behavior considering physiological differences between male and female subjects. This study investigated the effect of elevated baseline work rate on the VO_2_ kinetics and MFP in 10 male and 10 female healthy subjects. Subjects performed 4 transitions of very-heavy (VH) intensity cycling in a randomized order after unloaded (U-VH) or moderate (M-VH) exercise. Maximal isokinetic efforts (MIE) were performed before and after each condition at two different cadences (60 or 120 rpm). Whereas baseline VO_2_ and time constant (τ) were significantly higher in M-VH compared to U-VH, the fundamental amplitude and the VO_2_ slow component (VO_2_sc) were significantly lower in M-VH (*p* < 0.05) in both sexes. Blood lactate concentration ([La]) and rate of perceived exertion (RPE) were not influenced by condition or sex (*p* > 0.05). The MFP post-exercise was not significantly influenced by condition in both sexes and cadences (Δtorque for males: at 60 rpm in U-VH = 13 ± 10 Nm, in M-VH = 13 ± 9 Nm; at 120 rpm in U-VH = 22 ± 14 Nm, in M-VH = 21 ± 12 Nm; for females: at 120 rpm in U-VH = 10 ± 9 Nm, in M-VH = 12 ± 8 Nm; *p* > 0.05), with the exception that female subjects presented smaller decreases in M-UH at 60 rpm compared to U-VH (11 ± 13 vs. 18 ± 14 Nm, respectively, *p* < 0.05). There was no correlation between the decrease in torque production and VO_2_ kinetics parameters (*p* > 0.05). The alterations in VO_2_ kinetics which have been suggested to be linked to changes in motor unit recruitment after elevated baseline work rate did not reflect alterations in MFP and fatigue in both sexes.

## Introduction

Over the last decades, numerous studies have aimed to understand the physiological mechanisms underlying a loss of work efficiency or an increase in the O_2_ cost per unit of work during constant-load exercise above the gas exchange threshold (GET), i.e., the slow component of O_2_ uptake kinetics (VO_2SC_). Ever since, Poole et al. (1988, 1991) demonstrated that the mechanism explaining the VO_2_sc is likely within the exercising muscle. Thereafter, previous literature investigated motor unit recruitment and/or muscle fatigue without taking the lactate metabolism or the O_2_ consuming process outside the exercising limbs as a dominant mediator into consideration (Jones et al., 2011). Cannon et al. (2011) described muscle fatigue as an increased ATP cost of already recruited motor units, instead of the recruitment of less efficient muscle fibers, as the primary mechanisms explaining the VO_2SC_. Notwithstanding, Keir et al. (2016) showed a relationship between the VO_2SC_ and the time course of peripheral muscle fatigue (muscle torque production in response to electrically stimulated contractions) during high-intensity exercise. Moreover, Temesi et al. (2017) suggested that subjects with slower VO_2_ kinetics (i.e., higher τ-values) experienced more peripheral fatigue during very-heavy (VH) cycling exercise. However, this relationship between VO_2_ kinetics and muscle fatigue remains to be elucidated. Hopker et al. (2016) analyzed the effect of muscle damage on VO_2_ kinetics and suggested that locomotor muscle fatigue does not influence the kinetic response, i.e., τ or the VO_2SC_. Furthermore, de Souza et al. (2016) showed differences in the VO_2SC_ and τ despite a similar magnitude of muscle fatigue during VH cycling exercise. do Nascimento Salvador et al. (2018) demonstrated that prior cycling exercise decreased the VO_2SC_ behavior, but did not modify the time-course of muscle torque production in a subsequent VH cycling bout.

Previous literature showed alterations in VO_2_ dynamic when high-intensity exercise was immediately preceded by an elevated baseline (elevated VO_2_-values and/or elevated work rate) (Hughson and Morrissey, 1982; Wilkerson and Jones, 2006; Jones et al., 2008; Da Boit et al., 2014; Wüst et al., 2014). Lower amplitudes of the fundamental component and VO_2_sc and a slower time response (i.e., higher τ-values) can be found during high-intensity exercise when preceded by an elevated baseline compared to unloaded pedaling. It has been suggested that these changes may represent adjustments in the muscle O_2_ delivery and/or the muscle recruitment of motor units which are characterized by less mitochondrial content, lower metabolic efficiency and are positioned higher in the muscle recruitment hierarchy (i.e., type II fibers) (Bearden and Moffatt, 2001; Wilkerson and Jones, 2006). Dimenna et al. (2010) stated that it is not the elevated baseline VO_2_ *per se* that explains a slower VO_2_ kinetics, but the proportionally greater contribution of higher-order fibers to power production during transitions from an elevated baseline work rate. This greater contribution of type II fibers could alter the interaction between muscle efficiency and VO_2_ kinetics. Notably, the effect of elevated baseline on muscle force production (MFP) and its association with VO_2_ kinetics remains to be established.

To the best of our knowledge, no study investigated the relationship between VO_2_ kinetics and muscle fatigue with respect to the differences in pulmonary and neuromuscular capacities between male and female subjects. Females present smaller lung volumes, lower resting lung diffusion capacities and differences in O_2_ delivery, O_2_ extraction and blood flow (Mitchell et al., 1992; Harms, 2006; Murias et al., 2013; Dominelli et al., 2015). Lower muscle mass in females could be accompanied by a lower oxygen delivery and utilization. Reis et al. (2017) stated that the lower cardiac and respiratory capacities during exercise could reduce O_2_ delivery and utilization to the muscle, and consequently, lead to slower VO_2_ kinetics in females. Up to date, surprisingly little is known about the gender differences in VO_2_ kinetics (see Reis et al., 2017, for more details). Hunter () indicated that females exhibit less fatigue (loss of maximal torque) than males for dynamic fatiguing contractions when the velocity of contraction was controlled. Moreover, there are differences between sexes in the neuromuscular activation pattern of the quadriceps muscle (Clark et al., 2005), in muscle mass activated (greater in males) (Enoka and Duchateau, 2008; Hunter, 2014), muscle fiber type (greater proportional of type II in men) and gene expression and interactions with sex-specific hormones (Maher et al., 2009; Liu et al., 2010). Thus, it is reasonable to state that differences in sex could influence the MFP behavior and consequently, VO_2_ kinetics during cycling exercise. If the relationship between muscle fatigue and VO_2_ kinetics is “cause and effect,” then this relation should be sex-specific. Regarding to these differences in muscle fiber type and fatigue in men and women, investigating muscle fatigue at different cadences might provide further mechanistic insight. It is already known that proportionally more type II fibers are activated at 120 rpm and therefore, isokinetic cycling at 120 rpm reflects predominantly type II fiber fatigue in contrast to cycling at 60 rpm which fatigues predominantly type I fibers (Sargeant, 2007; Cannon et al., 2011). Moreover, understanding the mechanistic bases of the VO_2_sc will be crucial when designing interventions to enhance performance (Jones et al., 2011). A better understanding of strategies to speed-up the phase II τ or reduce the VO_2_sc and muscle inefficiency associated with it has the potential to increase exercise tolerance in both, male and female subjects.

Thus, the main aim of the present study was to investigate whether changes in VO_2_ kinetics after elevated baseline work rate reflect alterations in MFP behavior during maximal isokinetic efforts at different cadences (60 and 120 rpm). Besides, to study whether differences between sexes could influence these changes. In a case of a cause-effect relationship between VO_2_ kinetics and muscle fatigue it was expected a “mirror image” between MFP behavior and VO_2_ kinetics. We hypothesized that (1) the work-to-work transitions lead to a lower VO_2_sc amplitude and a slower time constant in both sexes; (2) female subjects present a lower amplitude of fundamental and slow phases compared to male counterparts; (3) changes in VO_2_sc and τ after elevated baseline work rate are accompanied by alterations in MFP, for both velocities and both sexes.

## Materials and Methods

### Ethics Statement

The present work was approved by the Research Ethics Committee of the Federal University of Santa Catarina and was conducted in accordance with the Declaration of Helsinki. After being fully informed of the risks and stresses associated with the study, the participants gave their written informed consent to participate.

### Participants

Twenty healthy participants (10 females: age 28 ± 6 years; mass 58 ± 7 kg; height 161 ± 5 cm; 10 males: age 26 ± 5 years; mass 75 ± 7 kg; height 177 ± 5 cm) volunteered to participate in the study. Subjects undertook exercise at a recreational level (3–4 sessions per week; 150–300 min per week), and were familiar with laboratory exercise testing procedures. Women performed the constant work-rate tests in the follicular phase being performed 2–4 days after the menses.

### Overview of Study Design

**Figure 1:**
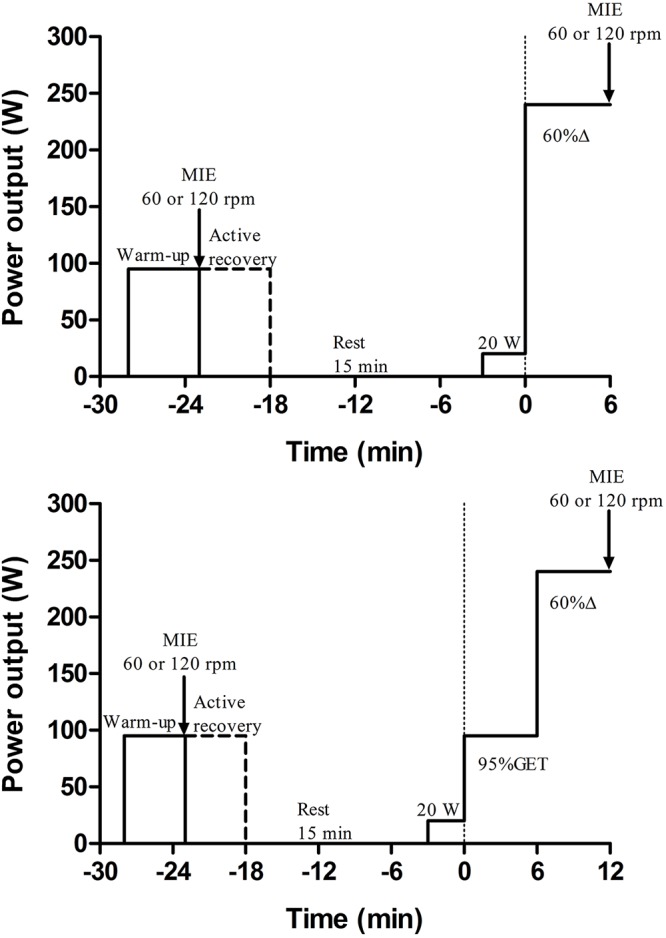
Experimental design for a representative participant. Top control condition (unloaded to very-heavy intensity exercise – U-VH); bottom experimental condition (moderate to very-heavy intensity exercise M-VH). Warm-up and active recovery were performed at 95% of the gas exchange threshold (GET). After a passive recovery period (15 min) participants started to cycling during 3 min of baseline (20 W) followed immediately by increases in the power output to 60% of the difference between the work-rate at the GET and VO_2peak_ (60% Δ; U-VH) or to 95%GET plus 60% Δ (M-VH). MIE, maximal isokinetic effort. Please note that M-VH was 6 min longer than U-VH (moderate + VH exercise).

Subjects were required to visit the laboratory on 5 occasions. On the first visit, each subject performed a maximal ramp test for the determination of the GET, VO_2peak_ and peak power output (P_peak_). On subsequent visits, subjects performed bouts of very heavy-intensity exercise immediately following unloaded (20 W – U-VH) or moderate (95% GET – M-VH) baseline to verify the effects of work-to-work transitions on VO_2_ kinetics and muscle force behavior (Figure 1). A maximal isokinetic effort (MIE; constant pedal cadence at 60 or 120 rpm) was performed following a standardized warm-up and immediately following the constant work-rate cycling bout to quantify reductions in peak torque. Subjects were instructed to avoid any intake of caffeine for 3 h, or alcohol and strenuous exercise in the 24 h preceding the test sessions and to arrive at the laboratory in a rested and fully hydrated state, at least 2 h post-prandial. All tests were performed at the same time of day in a controlled environmental laboratory condition (19–22°C; 50–60% RH) to minimize the effects of diurnal biological variation. Subjects performed only one test on any given day, and each test was separated by at least 48 h but completed within a period of 2 weeks.

### Equipment

All tests were performed on an electromagnetically braked cycle ergometer (Excalibur Sport PFM, Lode BV, Groningen, Netherlands). Respiratory and pulmonary gas exchange variables were measured using a breath-by-breath analyzer (Quark PFTergo, Cosmed, Rome, Italy). Before each test, the O_2_ and CO_2_ analysis systems were calibrated using ambient air (20.94% O_2_ and 0.03% CO_2_) and a gas of a known O_2_ and CO_2_ concentration (16.00% O_2_ and 5.00% CO_2_) according to the manufacturer’s instructions. Likewise, the turbine flow meter was calibrated before each test using a 3 L syringe (Quark PFTergo, Cosmed, Rome, Italy). A monitor connected to the gas analyzer was used to measure heart rate (HR). Capillary blood samples (25 μl) were obtained from the earlobe of each subject and the blood lactate concentration ([La]) was measured using an electrochemical analyzer (YSI 2700 STAT, Yellow Springs, OH, United States). The cycle ergometer, the breath-by-breath analyzer and the electrochemical analyzer were calibrated in accordance with specific manufacturer’s recommended procedures.

### Determination of GET and VO_2peak_

On the first laboratory visit, 15 min after the isokinetic sprint familiarization subjects performed an incremental ramp test for the determination of the GET, VO_2peak_, and P_peak_. After a 4 min period of cycling at 20 W (baseline), an incremental ramp test to exhaustion was undertaken with power output increasing by a rate of 30 W.min^-1^ from the baseline. Subjects were instructed to maintain their preferred cadence (female 77 ± 6 rpm; male 82 ± 6 rpm) throughout the test. The preferred cadence along with saddle and handle bar height and configuration was recorded and replicated in subsequent tests. Each subject was verbally encouraged to undertake maximal effort. The test was terminated when the cadence fell by more than 10 rpm below the preferred cadence for more than 5 s despite strong verbal encouragement (Black et al., 2015). Breath-by-breath pulmonary gas exchange and HR data were measured continuously during the test and averaged over 15 s periods. VO_2peak_ was defined as the highest value obtained in a 15 s interval, or if a VO_2_ plateau observed, it was considered as the average of the final minute of exercise (Day et al., 2003). The attainment of VO_2peak_ was defined using the criteria proposed by Bassett and Howley (2000). The P_peak_ was considered as the highest power output attained during the test. The GET was determined using a cluster of measurements as the V-slope method and the ventilatory equivalent method (Beaver et al., 1986). The data from the ramp test were used to calculate the work rate corresponding to 60% Δ (i.e., GET plus 60% of the difference between the work-rate at the GET and VO_2peak_). The lag in VO_2_ during incremental exercise taken into account by a deduction of two-thirds of the ramp rate from the work-rate at the GET (Burnley et al., 2011).

### Maximal Isokinetic Effort Measurement

The cycle ergometer was instrumented with pedal force measurements (Lode PFM, Groningen, Netherlands) to quantify muscle fatigue during the MIE. A switch from the hyperbolic mode to the isokinetic mode happened instantaneously when required. This protocol was similar to previously used protocols (Cannon et al., 2011; de Souza et al., 2016; Hopker et al., 2016; do Nascimento Salvador et al., 2018) and considered as reliable (do Nascimento Salvador et al., 2018) (no differences were observed for both peak torque – intraclass correlation coefficient = 0.99, typical error = 3.7% and, peak power output – intraclass correlation coefficient = 0.99; typical error = 4.2% obtained during the pre-exercise assessments). Peak torque was assessed for each subject by a 5 s cycling sprint test in the isokinetic mode at 60 or 120 rpm. In the pre-exercise muscle function assessment, subjects performed a 5 min warm-up at 95% GET immediately followed by the 5 s MIE. After this, subjects performed 5 min of active recovery at 95% GET and a period of 15 min rest before the main exercise bouts. The MIE was repeated immediately after the exercise in U-VH and M-VH (see Figure 1). Subjects were given an auditory cue to begin the all-out effort in the seated position and strong verbal encouragement was given throughout the 5 s. The torque and power data were recorded continuously during the MIE. As described by Altenburg et al. (2007), the peak torque in each crank arm was determined as the average of the four consecutive highest torque values (2 s). Thus, the peak torque during the MIE was then considered as the average of the peak values of both left and right crank arms.

### Data Analysis

Breath-by-breath data for each test were initially examined to exclude outlier values caused by sighs, swallowing and coughs (Lamarra et al., 1987). After that, for each exercise transition, the breath-by-breath data was processed and analyzed as described previously (do Nascimento et al., 2015; de Souza et al., 2016; do Nascimento Salvador et al., 2018). Non-linear regression techniques were used to fit the data after the onset of a fundamental phase with an exponential function (OriginPro 8; OriginLab). An iterative process ensured that the sum of squared errors was minimized. Due to large inter-individual differences in the duration of the exponential region (Murgatroyd et al., 2011), the identification of the VO_2_sc during the VH intensity exercise was performed individually. The fundamental VO_2_ kinetics (phase II) was isolated following the iterative method to identify the exponential region (Rossiter et al., 2001, 2002; Murgatroyd et al., 2011). The identification at the end of the fundamental phase (i.e., TD_s_) was performed by fitting a window from the start of the fundamental phase (i.e., after 20 s cardio-dynamic phase) initially set at 60 s. The window was lengthened iteratively until the exponential model fit demonstrated a discernible and consistent departure from the measured VO_2_-values by considering the criteria proposed in literature (Rossiter et al., 2001, 2002; Murgatroyd et al., 2011). Thus, the fitting window was constrained to this time point and a single-exponential fitting was performed only on the fundamental phase to identify the kinetics parameters. The model was constrained in VO_2baseline_ to aid in the identification of the key parameters according to the following equation:

(1) $${\text{VO}}_{\text{2}}\;\left(t\right)\;=\;{\text{VO}}_{\text{2baseline}}\;+\;Ax[\text{1}\;\text{-}\;e^{-\left(\frac{\text{t-TD}}{\tau }\right)}]$$

where: VO_2_(*t*) represents the value of VO_2_ at a given time (*t*); VO_2baseline_ is the average value over the last minute of baseline cycling; A is the asymptotic amplitude for the exponential term describing changes in VO_2_ from baseline to its asymptote; τ is the time constant; and the TD is the time delay. The VO_2SC_ was calculated according to the Equation (2):

(2) $${\text{VO}}_{\text{2}}\text{SC}\;=\;{\text{VO}}_{\text{2end}}\;\text{-}\;({\text{VO}}_{\text{2baseline}}\;+\;A)$$

Where: VO_2end_ is the average VO_2_ value over the last 20 s of a 6 min exercise bout.

### Statistical Analysis

Descriptive statistics are expressed as mean ± standard deviation. The Shapiro–Wilk test was applied to ensure a Gaussian distribution of the data (*n* < 50). A two-way mixed-model ANOVA was used to analyze the interaction over time and condition. Assumptions of sphericity were assessed using the Mauchly test, and any violation was corrected using the Greenhouse-Geisser correction factor. The Shapiro–Wilk test was used to verify the normality of residuals. When significant effects were observed the Bonferroni *post hoc* test was used for pairwise comparisons. Analyzes were performed using the Statistical Package for Social Sciences Windows (SPSS Inc. version 17.0; Chicago, IL, United States). The level of significance adopted was set at *p* < 0.05.

## Results

HR_max_, VO_2peak_, and P_peak_ were 183 ± 10 bpm, 46.8 ± 8.2 ml.kg^-1^.min^-1^ and 351 ± 51 W for males and; 181 ± 7 bpm, 39.2 ± 8.3 ml.kg^-1^.min^-1^, and 235 ± 26 W for females, respectively (Table 1). The [La] at the beginning and at the end of the ramp test were 1.4 ± 0.3 and 11.0 ± 2.5 mmol.L^-1^ for males and; 1.7 ± 0.6 and 9.1 ± 1.9 mmol.L^-1^ for females, respectively. The 60% Δ was performed at 240 ± 33 W and 157 ± 23 W and represented 82 ± 3 and 85 ± 3% of VO_2peak_ for male and female subjects, respectively.

**Table 1:** Anthropometric and maximal values during incremental test in male and female subjects.

Parameters	Male (*n* = 10) Mean ± SD (CI95%)	Female (*n* = 10) Mean ± SD (CI95%)
Age (years)	26.3 ± 4.7 (23–30)	28.4 ± 6.1 (24–33)
Body mass (kg)	75.1 ± 7.0 (70–80)	57.7 ± 6.6 (53–62)^∗^
Height (cm)	176.5 ± 4.8 (173–180)	160.8 ± 5.4 (157–165)^∗^
P_peak_ (W)	350.8 ± 50.7 (315–387)	234.9 ± 26.3 (216–254)^∗^
VO_2peak_ (L.min^-1^)	3.5 ± 0.6 (3.1–3.95)	2.2 ± 0.4 (1.95–2.53)^∗^
VO_2peak_ (ml.kg.min^-1^)	46.8 ± 8.2 (41–53)	39.2 ± 8.3 (33–45)^∗^
HR_max_ (bpm)	183 ± 10 (176–190)	181 ± 7 (176–186)
VE_max_ (L.min^-1^)	167.2 ± 35.7 (142–193)	94.4 ± 20.5 (80–109)^∗^
GET (ml.kg.min^-1^)	25.8 ± 4.7 (22–29)	24.3 ± 4.7 (21–28)
GET (W)	129 ± 20 (114–143)	95 ± 14 (86–105)^∗^

*^∗^Difference between sexes p < 0.05. P_*peak*_, peak power; VO_*2peak*_*, *VO_*2*_ peak values; HR_*max*_*, *maximal heart rate; VE_*max*_*, *maximal minute ventilation; GET, gas exchange threshold.*

### Square-Wave Exercise Bouts

**Figure 2:**
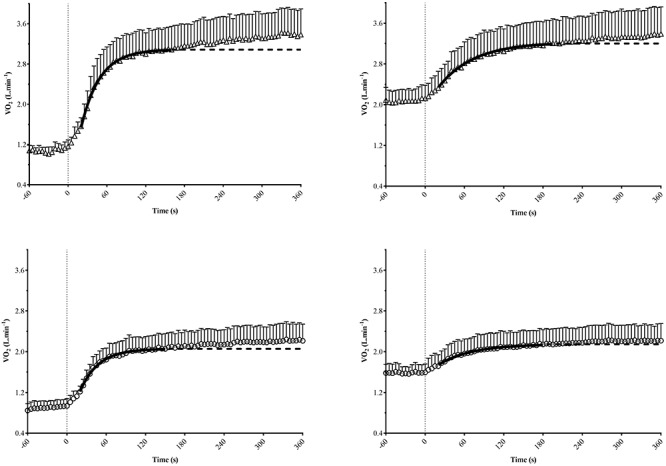
Mean group values of oxygen uptake (VO_2_) kinetics for male (top) and female (bottom) subjects during transitions from unloading (left) or moderate (right) exercise. Non-linear least-squares regression modeling (continuous black line), with the fit extrapolated (dashed line) to the end of exercise were showed. Standard deviation values were shown just on the upper side of mean symbols for clarity.

**Figure 3:**
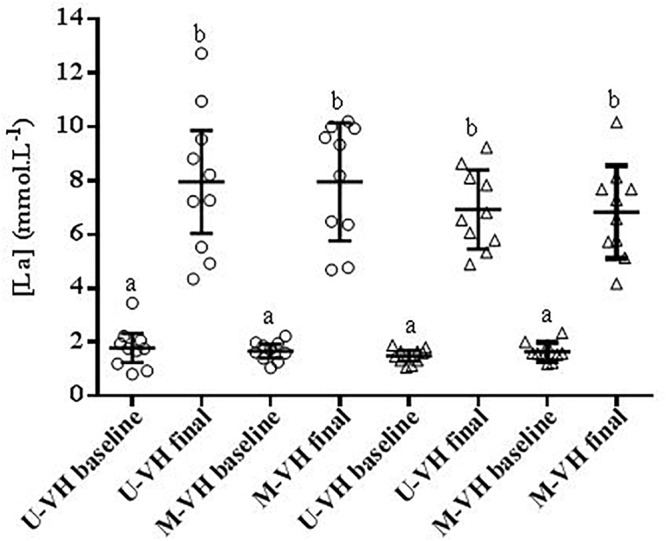
[La] = blood lactate concentration. Circle symbols show the female values. Triangle symbols show male values. U-VH, unloaded to very heavy intensity exercise; M-VH, moderate to very heavy intensity exercise. Different letters showed significant differences *p* < 0.05.

**Figure 4:**
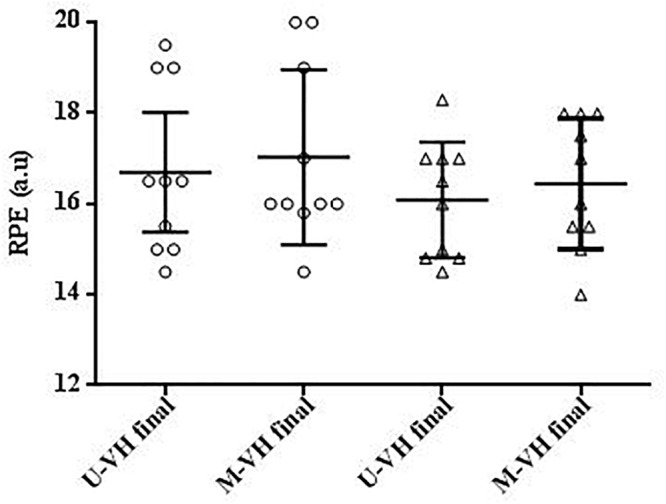
RPE = rating of perceived exertion. Circle symbols show female values. Triangle symbols show male values. U-VH, unloaded to very heavy intensity exercise; M-VH, moderate to very heavy intensity exercise.

During M-VH, the VO_2baseline_ was significantly higher, A, TD, and VO_2sc_ lower and τ and TDs slower compared to U-VH for both sexes (*p* < 0.05). There were no significant differences for Atotal (i.e., A + VO_2baseline_) and VO_2end_ between conditions for both sexes (*p* > 0.05; Table 2). Female subjects presented lower amplitudes, VO_2sc_ and VO_2end_ than male counterparts (*p* > 0.05; Figure 2). The [La] post-exercise was not significantly different between conditions or sexes (*p* > 0.05; Figure 3). There was no difference between conditions (*p* = 0.23) in both sexes (*p* = 0.31) for rate of perceived exertion (RPE), thus, male and female subjects perceived the effort in a similar way after the exercise in both conditions (Figure 4).

**Table 2:** VO_2_ kinetics responses during rest-to-work and work-to-work exercise in male and female subjects.

	Male		Female	
	U-VH	M-VH	U-VH	M-VH
Parameters	Mean ± SD (CI95%)		Mean ± SD (CI95%)	
VO_2baseline_ (L.min^-1^)	1.0 ± 0.1 (1.0–1.2)	2.1 ± 0.2^∗^ (1.9–2.2)	0.9 ± 0.1 (0.8–1.0)	1.6 ± 0.2^∗^^ 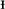 ^ (1.5–1.7)
A (L.min^-1^)	2.0 ± 0.4 (1.7–2.2)	1.1 ± 0.3^∗^ (0.9–1.3)	1.1 ± 0.3bbb (0.9–1.4)	0.5 ± 0.2^∗^^ 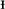 ^ (0.4–0.7)
A_TOTAL_ (L.min^-1^)	3.1 ± 0.4 (2.8–3.4)	3.2 ± 0.4 (2.9–3.5)	2.0 ± 0.3^ 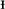 ^ (1.9–2.2)	2.1 ± 0.3^ 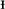 ^ (1.9–2.3)
VO_2SC_ (L.min^-1^)	0.30 ± 0.14 (0.20–0.40)	0.18 ± 0.14^∗^ (0.08–0.29)	0.18 ± 0.10^ 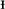 ^ (0.10–0.25)	0.09 ± 0.07^∗^^ 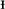 ^ (0.04–0.14)
τ (s)	28.8 ± 8.5 (22.7–34.9)	54.9 ± 22.4^∗^ (38.8–71.0)	27.4 ± 5.3 (23.6–31.2)	48.3 ± 19.0^∗^ (34.8–61.9)
TD (s)	13.9 ± 4.2 (10.9–16.9)	07.3 ± 6.1^∗^ (02.9–11.7)	11.0 ± 6.2 (6.6–15.4)	04.8 ± 8.1^∗^ (00.0–10.7)
TD_S_ (s)	164 ± 27 (144–183)	195 ± 44^∗^ (164–227)	154 ± 27 (134–173)	179 ± 48^∗^ (145–214)
VO_2END_ (L.min^-1^)	3.4 ± 0.5 (3.0–3.7)	3.4 ± 0.5 (3.0–3.8)	2.2 ± 0.3^ 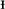 ^ (2.0–2.5)	2.2 ± 0.3^ 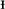 ^ (2.0–2.4)

*^∗^Differences between conditions p < 0.05. ^
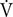
^Differences between sex within condition p < 0.05. U-VH, unloaded very-heavy; M-VH, moderate very-heavy****;****A, amplitude; Atotal, amplitude + VO_2baseline_; VO_2sc_*, *VO_2_ slow component; τ, time constant; TD, time delay, TDs, TD of slow component phase; VO_2end_*, *VO_2_ at the end of exercise bout.*

#### Torque Production Behavior

**Figure 5:**
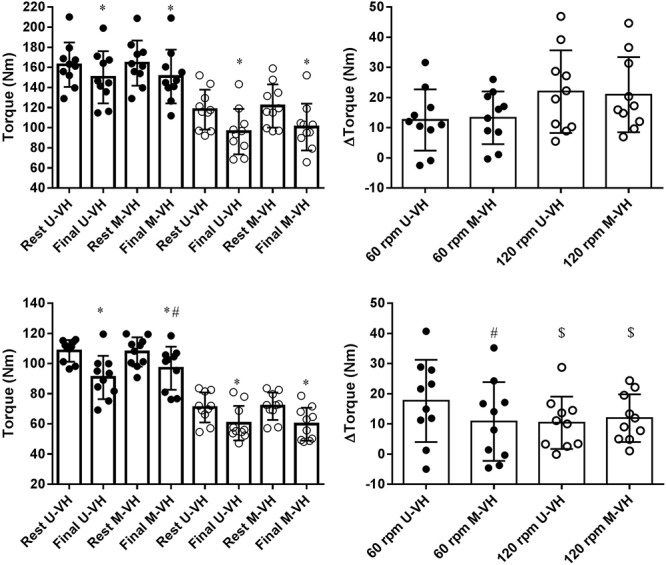
Torque production behavior during maximal isokinetic efforts at 60 (black circles) or 120 (open circles) rpm for males (top panels) or females (bottom panels) subjects. Right panels show Δtorque values. U-VH, unloaded to very heavy intensity exercise; M-VH, moderate to very heavy intensity exercise. ^$^Differences between sex within velocity *p* < 0.05. ^∗^Differences within condition *p* < 0.05. ^#^Differences between conditions *p* < 0.05.

There were no significant differences in MFP between conditions (U-VH vs. M-VH) for men in both velocities (60 rpmml: main effect *condition* vs. *time*, *F* = 0.09; *p* = 0.77; 120 rpmml: main effect *condition* vs. *time*, *F* = 0.48; *p* = 0.50). Female subjects showed no significant differences in MFP between conditions at 120 rpm (main effect *condition* vs. *time*, *F* = 0.36; *p* = 0.56), but smaller decreases in torque production were observed following M-VH at 60 rpm compared to U-VH (main effect *condition* vs. *time*, *F* = 18.2; *p* = 0.01). The torque decrement at 120 rpm was lower for female than male subjects (*p* < 0.05, Table 3 and Figure 5). There were no significant correlation between Δ torque and VO_2SC_ or τ in the different conditions or velocities in both sexes.

**Table 3:** Torque production (Nm) behavior before and after rest-to-work and work-to-work exercise in male and female subjects.

		Unloaded very-heavy Mean ± SD (CI95%)		Moderate very-heavy Mean ± SD (CI95%)	
		Initial	Final	Initial	Final
Male	60 rpm	163 ± 22	150 ± 26^∗^	164 ± 22	151 ± 27^∗^
		(147–179)	(132–169)	(148–180)	(132–170)
	120 rpm	118 ± 20	96 ± 23^∗^	122 ± 22	101 ± 23^∗^
		(104–132)	(80–112)	(106–137)	(84–117)
Female	60 rpm	108 ± 7	91 ± 14^∗^	108 ± 10	97 ± 14^∗#^
		(103–114)	(81–101)	(101–115)	(87–107)
	120 rpm	71 ± 10	60 ± 11^∗^	72 ± 9	60 ± 11^∗^
		(64–78)	(52–69)	(65–78)	(52–68)

*^
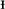
^ Differences between sex within velocity p < 0.05. ^∗^Differences within condition p < 0.05. ^#^Differences between conditions p < 0.05.*

## Discussion

The main finding of this study was that alterations in VO_2_ kinetics induced by preceding elevated baseline work rate did not reflect alterations in MFP for both healthy males and females. Thus, this work has demonstrated experimentally that isolated decreases in VO_2_sc and fundamental phase amplitudes or changes in TDs and τ are not linked to MFP during high-intensity cycling exercise in healthy male and female subjects despite the differences between sexes. Considering that there were no differences between conditions (U-VH vs. M-VH) or sexes in RPE and [La], it is suggested that the effects of work-to-work transitions on the VO_2_ kinetics can be dissociated from differences in blood acidosis and the perception of effort. We hypothesized that elevated baseline work rate would lead to lower amplitudes of the VO_2SC_ and slower values for τ for both sexes despite of the differences between male and female counterparts. Our findings confirm these hypotheses. However, the hypothesis stating that changes in VO_2_ kinetics would be accompanied by alterations in MFP for both velocities and both sexes was not confirmed.

### The Effects of Elevated Baseline Work Rate on VO_2_ Kinetics

The putative mechanism likely explaining the alterations in VO_2_ dynamics preceded by elevated baseline VO_2_ may be represented by the balance between the parasympathetic and sympathetic control of the HR. Elevated work rates seem to alter parasympathetic withdrawal leaving the slower sympathetic control to mediate increases in HR (Hughson and Morrissey, 1982; Bearden and Moffatt, 2001; DiMenna et al., 2010). It has been suggested that a slowing of the HR kinetics may limit the O_2_ delivery to cellular respiration (Hughson and Morrissey, 1982; DiMenna et al., 2010). Alternatively, cellular respiration might adjust more slowly in muscle fibers that are already active and/or recruitment of motor units that are believed to possess slower VO_2_ kinetics and a higher VO_2_ cost of tension (Poole et al., 2008). Likewise, the fundamental PCr τ is lengthened and the fall in PCr is greater compared to U-VH transitions (Jones et al., 2008). These mechanisms are consistent with a greater proportional involvement of muscle fibers that are positioned higher in the recruitment hierarchy (e.g., type II muscle fibers) (Henneman et al., 1965; Jones et al., 2008; Dimenna et al., 2010). Although the present study does not allow to distinguish between the effect of elevated VO_2_ and elevated work-rate (Please, see the Supplementary Material about this discussion), our results extend the effects of elevated baseline work rate from previous literature for female subjects (Hughson and Morrissey, 1982; Wilkerson and Jones, 2006; Jones et al., 2008; Dimenna et al., 2010; Da Boit et al., 2014; Wüst et al., 2014).

It is important to acknowledge that training status largely influences 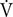
O_2_ kinetics, presenting higher phase II τ-values in untrained vs. trained subjects (Koppo et al., 2004). Our phase II τ-values are similar compared with participants from similar training status (recreationally active) at similar intensities (60% Δ) (DiMenna et al., 2010; Cannon et al., 2011; Da Boit et al., 2014; Keir et al., 2016). Despite the differences in the amplitudes of the fundamental and slow phases between sexes in both U-VH and M-VH, τ was not significantly different between sexes despite an elevated baseline. These results are in contrast to previous findings reported for τ in adolescents (Fawkner and Armstrong, 2003; Lai et al., 2016), but in agreement in relation to VO_2SC_. Besides, we extend these results to a healthy population during cycling compared to the results of Reis et al. (2017) which showed no differences in τ between women and men trained swimmers during heavy-intensity swimming. Moreover, our results are in accordance to findings reported for middle aged subjects during moderate cycling (O’Connor et al., 2012). To date, no study has investigated the effect of work-to-work transition on the VO_2_ kinetics and MFP comparing sexes. Although males present higher fundamental amplitudes and similar τ-values compared to females, the gross rate of increase of oxygen uptake per second is higher in men, suggesting a quicker onset (Reis et al., 2017). According to Reis et al. (2017) this could be due to the higher VO_2peak_ and anatomic differences (e.g., muscle mass) presented by males. It has been reported that females could present lower O_2_ delivery, O_2_ extraction and blood flow due to smaller hearts, smaller lung volumes and lower diffusion capacities and cardiac outputs (Harms, 2006; Dominelli et al., 2015). The present study found significant lower values for VO_2peak_, power at GET and VE for females compared to their male counterparts. This could be a result of the anatomic differences in the cardiorespiratory system. According to Murias et al. (2013) females were less effective in matching O_2_ delivery and O_2_ utilization, which may be due to a lower tonic sympathetic activity in women leading to systemic differences in blood flow distribution. However, a lower oxygen delivery to the muscles seems not to influence the VO_2_ kinetics in high-intensity exercise (Reis et al., 2017). Olfert et al. (2004) affirmed that female subjects did not experience greater O_2_ diffusion limitations during exercise, which may emphasize the importance of absolute lung size or aerobic fitness in determining susceptibility to gas exchange impairment rather than sex *per se*.

### VO_2_ Kinetics vs. Muscle Force Production

Previous literature demonstrated a relation between VO_2_ kinetics parameters (i.e., VO_2SC_, τ) and the loss in torque/force production during high-intensity exercise (Cannon et al., 2011; Keir et al., 2016; Temesi et al., 2017). Cannon et al. (2011) proposed that greater levels of muscle fatigue are reflected by a larger amplitude of the VO_2SC_ or vice-versa. The mechanisms contributing to peripheral muscle fatigue has been suggested to contribute to an increased O_2_ cost of exercise (Keir et al., 2016). Moreover, Temesi et al. (2017) suggested that subjects with slower VO_2_ kinetics (higher τ-values) experience a greater level of peripheral muscle fatigue, specifically, more excitation-contraction coupling failure. Thus, it was expected that alterations of MFP behavior would be displayed in the VO_2_ kinetics. In a case of cause-effect relationship, it was expected that higher τ-values would be related with a greater muscle force loss. Or in other hand, lower VO_2SC_ would be related with a lower muscle force decrement. However, we could not confirm this hypothesis. The decrease in torque production in both conditions and for both sexes were not related or linked with the VO_2SC_ or τ. Our results are in accordance with Hopker et al. (2016) who showed that exercise-induced muscle damage led to significant locomotor muscle fatigue, but did not alter the VO_2SC_ or τ during subsequent high-intensity cycling. Hopker et al. (2016) also affirmed that the results from Cannon et al. (2011) could be misleading because the relationship presented by these authors was considering different intensity domains. Deley et al. (2006) demonstrated an inverse relation between the level of muscle fatigue in type II muscle fibers induced by an electromyostimulation protocol and the VO_2SC_ during subsequent high-intensity cycling. Additionally, τ was not altered by this intervention. Moreover, Thistlethwaite et al. (2008) investigated two types of prior exercise (knee extension vs. cycling) which caused different activation patterns/levels of additional motor units (∼38% in knee extension vs. 21% in cycling) and found similar VO_2_ responses (VO_2SC_ or τ) during the subsequent bout of heavy cycling exercise. The authors concluded that muscle fatigue is neither the primary determinant of the VO_2SC_ nor does it affect the τ of the fundamental phase. Recently, do Nascimento Salvador et al. (2018) showed that prior VH cycling changed the VO_2SC_ and the trajectory of the VO_2SC_ in a subsequent VH cycling exercise, but did not alter MFP behavior. The present study is in line with these findings and challenges a “cause-effect” relationship between VO_2SC_ and muscle fatigue.

The present study observed no differences in Δtorque at 60 and 120 rpm following M-VH and U-VH for males and at 120 rpm (Figure 5) for females despite the differences in the VO_2_ kinetics. The differences in VO_2_ fundamental and slow amplitudes as well as in muscle torque production in absolute values could indicate a possible link between muscle force and VO_2_ kinetics and this hypothesis was not discarded. However, no correlation between Δtorque and VO_2SC_ or τ was found in any condition or velocity in both sexes. These results do not support previous literature proposing a causative relation between muscle fatigue and VO_2_ kinetics (Cannon et al., 2011; Keir et al., 2016; Temesi et al., 2017). However, female participants showed a smaller decrement in torque production in M-VH at MIE 60 rpm. This may be explained by a smaller percentage of type II fibers and a greater fatigue resistance in females compared to males (Maher et al., 2009; Liu et al., 2010; Hunter, 2014). Further, a reduced proportion of type II muscle fibers may explain the smaller decrements in torque production by females observed at 120 rpm at which the reliance on type II muscle fibers increases. Considering the higher percentage of type II muscle fibers in men (Maher et al., 2009), it may be suggested that proportionally more type II fibers were activated at 120 rpm (Sargeant, 2007) and consequently, led to a greater level of muscle fatigue compared to 60 rpm and compared to women. When exercise is preceded by an elevated baseline work rate, it may be suggested that the recruitment of additional motor units which are characterized by a smaller mitochondrial content and a higher VO_2_ cost per unit of force (i.e., type II fibers) becomes inevitable in order to maintain the exercise intensity (Wüst et al., 2014). Thus, female subjects may have shown a lower Δtorque in M-VH at 60 rpm because of a lesser reliance on type II fibers.

It is noteworthy that Temesi et al. (2017) did not find a relationship between changes in maximal voluntary activation and τ-values. They stated a lack of relationship between τ-values and central fatigue. According to Hopker et al. (2016), the RPE measured indirectly supports the assumption that a level of central motor command was required in order to produce the power output. This study found that RPE immediately post-exercise was neither different between conditions (U-VH vs. M-VH) nor sexes. Based on these considerations, a dissociation between the changes in VO_2_ kinetics (i.e., slower τ and the lower VO_2SC_) and a decrease in central motor drive may be suggested. The performance of a maximal isokinetic cycling effort taken immediately post-exercise is a “global” but ecologically valid protocol to quantify the decrease in MFP in cycling. However, the identification of the origin of fatigue (i.e., central or peripheral) is not possible. Beelen et al. (1995) suggest that fatigue in this type of dynamic isokinetic exercise may be due to changes in the muscle itself and not due to failure of central drive. Considering that muscle fatigue measured by MIE recovers back to baseline values within 1–3 min (Beelen et al., 1995; Coelho et al., 2015; do Nascimento Salvador et al., 2018), it represents a tendency to indicate more the peripheral fatigue.

## Conclusion

In summary, this investigation has demonstrated that the fundamental and the VO_2SC_ amplitude were smaller and the time constant and time delay of the slow phase were longer during high-intensity cycling exercise from elevated baseline work rate despite of the differences in both sexes. These alterations were dissociated from the changes in blood lactate concentration and from the perception of exertion. The same RPE for both sexes in both conditions may indicate a similar level of fatigue in central motor drive. Further, MFP decreased following exercise to a similar magnitude for both conditions (U-VH and M-VH) in males and females, with the exception that females demonstrated a smaller decrease following M-VH during isokinetic efforts at 60 rpm compared to U-VH. This pattern in torque production could be related to the activation pattern and distribution of muscle fiber type in men and women. The decrease in muscle force was not associated with VO_2_ kinetics parameters. Thus, isolated alterations in VO_2_ kinetics after work-to-work transitions, which may be linked to changes in motor unit recruitment, do not reflect alterations in MFP and fatigue in healthy male and female subjects. These results challenge a “cause-effect” relationship between VO_2_sc or τ and muscle fatigue.

## Ethics Statement

The present work was approved by the Research Ethics Committee of the Federal University of Santa Catarina and was conducted in accordance with the Declaration of Helsinki. After being fully informed of the risks and stresses associated with the study, the participants gave their written informed consent to participate.

## Author Contributions

BD and PdNS conceived and designed the work. PdNS, BG, and BD acquired, analyzed, and interpreted the data for the work. PdNS, LS, BG, LG, and BD drafted the work or revised it critically for important intellectual content. All authors have approved the final version of the manuscript and agreed to be accountable for all aspects of the work. All persons designated as authors qualify for authorship, and all those who qualify for authorship are listed.

## Conflict of Interest Statement

The authors declare that the research was conducted in the absence of any commercial or financial relationships that could be construed as a potential conflict of interest.
