# Supplementary material for: Changes in VO2 Kinetics After Elevated Baseline Do Not Necessarily Reflect Alterations in Muscle Force Production in Both Sexes
Source: Front Physiol. 2019 Apr 25;10:471. doi: 10.3389/fphys.2019.00471 (PMC6495266; doi:10.3389/fphys.2019.00471)
Supplement: Supplementary file 1 [file Table_1.docx]

*There would still be a difference in τ between conditions when the V̇O_2_-kinetics in U-VH were modeled from the V̇O_2_ value that matches the baseline V̇O2 of M-VH*

It is important to note that the effects of priming exercise as seen in the literature have to be distinguished from elevated baseline effects. Heavy priming exercise (but not moderate) typically increases phase II amplitude and decreases V̇O_2_sc and mean response time, without effects on time constant of phase II. Meanwhile, when a high-intensity exercise bout is immediately preceded by an elevated work rate (usually moderate exercise), lower A and longer τ values can be found. It is neither a fit artefact nor something related to the adjustments. As previously reported by others (Dimmenna et al. 2010 - DOI: 10.1016/j.resp.2010.04.017; Jones et al. 2008 - DOI: 10.1113/jphysiol.2007.142026), it is related to the balance between the parasympathetic and sympathetic control of the HR. a limited O_2_ delivery to cellular respiration, slower cellular respiration in already active fibers and greater proportional involvement of type II muscle fibers. In our opinion, there is no sense to modeled U-VH with the baseline from M-VH, it is not the physiological reality. Despite, we analyzed for some subjects fixing the V̇O_2_ baseline from M-VH in U-VH, τ values were unchangeable and what is increasing in this case is TD, as expected.

*Mechanistic alterations in muscle fiber activation due to elevated baseline.*

The evidence in the literature basically show that when a VH bout of exercise is preceded by a moderate exercise, which happens is the muscle recruitment of motor units which are characterized by less mitochondrial content, lower metabolic efficiency and are positioned higher in the muscle recruitment hierarchy (i.e. type II fibers). More details in Jones et al. (2008 - DOI: 10.1113/jphysiol.2007.142026).

*Changes in V̇O_2_ kinetics that logically follow from starting at a higher V̇O_2_ in the M-HV condition. Given the linear relationship between V̇O_2_ and PO, automatically, A is smaller and τ larger in the M-HV condition.*

These mechanistic alterations in muscle fiber activation are linked to V̇O_2_ kinetics and after an elevated baseline we observe a lower fundamental amplitude and a slower phase II time constant. Dimmenna et al. (2010 - DOI: 10.1016/j.resp.2010.04.017) showed that is not the elevated baseline per se which change the V̇O_2_ kinetics (They used a protocol with a high-intensity exercise preceded by a high-intensity bout and a short period of recovery = higher V̇O_2_ baseline) but is the elevated work rate. That means a different muscle recruitment behavior. Jones et al. (2008 - DOI: 10.1113/jphysiol.2007.142026) showed a link between PCr dynamics and elevated baseline work rate.

In relation to the fit constrained at baseline from M-VH condition, for us there is no physiological sense to do that. If the case we would use a baseline value from a different condition to analyze the U-VH condition. We aimed to see the effects of elevated baseline on V̇O_2_ kinetics and muscle force behavior; we started the study considering the existing evidence in the literature about these effects. Mathematically speaking when we constrain the data in a different value from the curve shape the adjust becomes weak. Sometimes the r^2^ is below 0.9 (that for kinetics is a mess) and the residuals sum of squares are larger. Despite, we are showing this kind of fit (U-VH constrained at baseline from M-VH condition) for some subjects and compare with the fit already done.

|  | U-VH condition | | | | |  | | | |
| --- | --- | --- | --- | --- | --- | --- | --- | --- | --- |
|  | **Fit already done** | | | | | **U-VH constrained at baseline from M-VH condition** | | | |
|  | **Baseline** | **A** | **τ** | **TD** |  | **Baseline** | **A** | **τ** | **TD** |
| Female | | | | |  |  | | | |
| F01 | 838.8 | 1385.1 | 20.0 | 16.4 |  | 1550.7 | 673.1 | 20.0 | 30.9 |
| F07 | 1177.3 | 941.4 | 32.9 | 17.7 |  | 1577.5 | 541.2 | 32.9 | 35.9 |
| F11 | 869.7 | 1387.6 | 23.3 | 12.5 |  | 1649.8 | 607.5 | 23.3 | 31.7 |
| F15 | 951.3 | 842.3 | 33.4 | 0.0 |  | 1372.7 | 432.5 | 40.8 | 19.4 |
| F16 | 861.5 | 1362.6 | 25.3 | 14.9 |  | 1763.3 | 460.3 | 25.3 | 42.4 |
| Male | | | | | | | | | |
| M14 | 1021.4 | 2072.5 | 24.9 | 13.1 |  | 2179.2 | 914.7 | 24.9 | 33.5 |
| M05 | 1170.6 | 2677.6 | 19.2 | 16.4 |  | 2385.2 | 1463.0 | 19.2 | 28.0 |
| M12 | 1037.2 | 1637.8 | 31.6 | 17.1 |  | 1914.4 | 760.6 | 31.6 | 41.4 |
| M20 | 1075.4 | 2037.2 | 19.6 | 20.6 |  | 2313.7 | 798.9 | 19.6 | 39.0 |
| M23 | 1047.0 | 1594.6 | 35.1 | 11.5 |  | 1904.0 | 737.6 | 35.1 | 38.6 |
