# Supplementary figures and images for: Changes in VO2 Kinetics After Elevated Baseline Do Not Necessarily Reflect Alterations in Muscle Force Production in Both Sexes
Source: Front Physiol. 2019 Apr 25;10:471. doi: 10.3389/fphys.2019.00471 (PMC6495266; doi:10.3389/fphys.2019.00471)

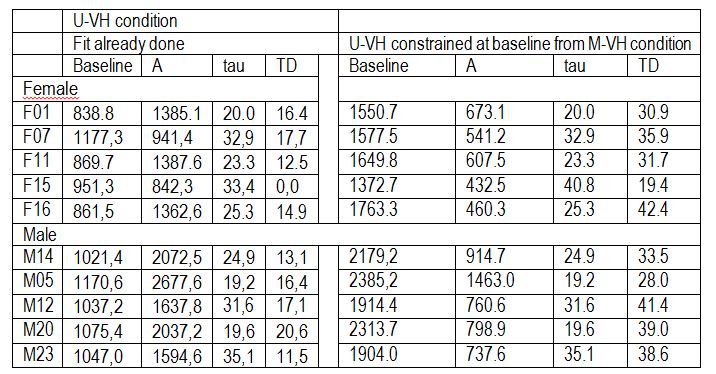

Supplement: Supplementary file 2 [file Image_1.JPEG]
